# Mathematical Model for Optimal Control of Soil-Transmitted Helminth Infection

**DOI:** 10.1155/2020/6721919

**Published:** 2020-08-01

**Authors:** Aristide G. Lambura, Gasper G. Mwanga, Livingstone Luboobi, Dmitry Kuznetsov

**Affiliations:** ^1^School of Computational and Communication Science and Engineering, The Nelson Mandela African, Institution of Science and Technology, P.O. Box 447, Arusha-, Tanzania; ^2^Department of Computer Systems and Mathemmatics, Ardhi University, P.O. Box 35176, Dar es Salaam, Tanzania; ^3^Department of Physcics, Mathematics and Informatics, University of Dar es Salaam, P.O. Box 2329, Dar es Salaam, Tanzania; ^4^Institute of Mathematical Sciences, Strathmore University, P.O. Box 59857-00200, Nairobi, Kenya

## Abstract

In this paper, we study the dynamics of soil-transmitted helminth infection. We formulate and analyse a deterministic compartmental model using nonlinear differential equations. The basic reproduction number is obtained and both disease-free and endemic equilibrium points are shown to be asymptotically stable under given threshold conditions. The model may exhibit backward bifurcation for some parameter values, and the sensitivity indices of the basic reproduction number with respect to the parameters are determined. We extend the model to include control measures for eradication of the infection from the community. Pontryagian's maximum principle is used to formulate the optimal control problem using three control strategies, namely, health education through provision of educational materials, educational messages to improve the awareness of the susceptible population, and treatment by mass drug administration that target the entire population(preschool- and school-aged children) and sanitation through provision of clean water and personal hygiene. Numerical simulations were done using MATLAB and graphical results are displayed. The cost effectiveness of the control measures were done using incremental cost-effective ratio, and results reveal that the combination of health education and sanitation is the best strategy to combat the helminth infection. Therefore, in order to completely eradicate soil-transmitted helminths, we advise investment efforts on health education and sanitation controls.

## 1. Introduction

The worldwide burden of helminth (worm) infection cannot be underestimated. It is estimated that over 1.5 billion people worldwide are infected with helminths [1]. Soil-transmitted helminths are among the NTDs which mostly affect the poorest and the resource-constrained populations of the world. Soil-transmitted helminths are caused by interstinal parasitic nematode hookworm, Ascaries lumbriocoides and Trichuris trichiura species through ingesting eggs in unwashed undercooked vegetables and unpeeled fruits in contaminated water sources or ingestion of eggs by children who play in contaminated environment. More than 267 million preschool-aged children and more than 568 million school-aged children (SAC) live in endemic areas where these parasites are highly transmitted. This establishes chronic infections over time which hinders and impairs the developmental and cognitive growth of both preschool- and school-aged children which in turn affect their academic performance. It also has social and economic deficits [2]. The possibility of elimination of the helminth infection is by reducing the number of worms/parasites in a host, but this cannot be achieved without application of other means of reducing the density of parasites from the contaminated environment. Hence proper treatment of the helminth infection and effective control strategy that target this group and infected adults is needed. Currently, there are control programmes to combat the disease which include regular treatment by mass drug administration (MDA), water sanitation, and health education. To reduce the morbidity of the soil-transmitted helminth, antihelminthic drugs like albendazole and benzimidazole have been used for decades but still there has not been an optimal application of the existing control measures with these treatment alternatives in endemic areas [3, 4].

Over years, mathematical models have helped to improve our understanding of population dynamics and provide tools for the assessment and evaluation of a number of control programmes in place. The mathematical model for the helminth infection can be traced back to the papers by [4–6]. Deterministic mathematical models inform policymakers to realize the effort required to increase control coverage for the achievement of less than one percent prevalence and intensity of soil-transmitted helminths by 2020 [7]. These models have been used intensively but none have applied optimal control for the soil-transmitted helminth infection.

However, optimization of the existing interventions tools for the helminth infection has been the priority research areas to examine their impact and sustainability [8]. The study on the implementation of these control measures and how to deliver them optimally is of great importance. Thus, in this study, we consider optimal control of the helminth mathematical model with the preventive measures (health education) to sensitize the susceptible population and treatment by mass drug administration and sanitation. The preventive measures include provision of education materials and educational messages and sanitation include proper provision of clean water, personal hygiene, and installation of public toilets while MDA is administered to the entire population (pre-SAC and SAC), but for the healthy individuals (susceptible and recovered), this treatment procedure becomes placebo.

## 2. Materials and Methods

### 2.1. Model Formulation

For modeling, the total human population is divided into four subpopulations *S*(*t*), *E*(*t*), *I*(*t*), and *R*(*t*). *S*(*t*) represents the number of individuals who are not infected but can be infected by helminth parasites. *E*(*t*) represents the number of individuals exposed to helminth infection but not infectious. *I*(*t*) represents the number of individuals infested with parasitic worms. *R*(*t*) represents the number of individuals recovered from the helminth infection. *M*(*t*) represents the parasite population in the environment. Individuals in the susceptible class contract infection after a sufficient contact with the contaminated environment. All the newborns enter a susceptible class at the rate *b* and are moved to the exposed class at the rate *λ*. Individuals in the exposed class are being reinfected as they interact with contaminated environment but remain latent for a period of 1/*ρ*, where *ρ* is the progression rate from the exposed to infective class. Again, we assume that an infected individual contaminate the environment at rate *ε* but do not acquire new infection; hence, individuals in the infected class are moved to the recovered class as the result of natural immunity at the rate *q*. Also, we assume that the recovered individuals are aware of infection so that individuals in this class may not interact with contaminated environment; furthermore, they have temporary immunity against the infection. Hence, individuals in the recovered class return to the susceptible class at a rate *γ* due to loss of immunity and awareness.

**Figure 1:**
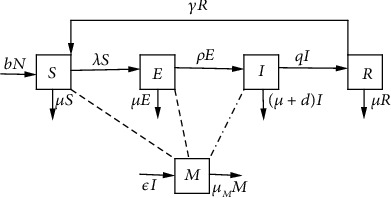
Compartmental diagram for the helminth model. The dashed lines indicate the interaction of human population with contaminated environment to ingest parasitic worms while the dashed line with dot indicate the rate at which individuals infested with helminths releases eggs to contaminate the environment.

The infected individuals contaminate the environment at the rate *ε* as they defecate outside the latrines to increase the concentration of parasitic worms in the soil. The parasitic worms in the soil infect human population at the rate

(1) $$\begin{array}{c} \lambda =\frac{\beta M}{K+M}, \end{array}$$

where *β* is the intake rate of eggs in contaminated food or larvae that has penetrated the skin to lead the transmission of infection and *K* is the number (density) of the helminths at which the infection rate is half the maximum rate. The natural mortality in each human class is denoted by *μ*, and the infectious individuals have added the helminth-induced death rate denoted by *d*. Further, we assume that the parasitic worms die naturaly at the rate *μ*_*M*_. The aforementioned assumptions and explanation give the dynamics of the helminth model as illustrated in Figure 1. Model parameters are fully described in Table 1. The model has five nonlinear ordinary differential equations given by system (2).

(2) $$\begin{array}{c} \frac{dS}{dt}=bN+\gamma R-\left(\mu +\lambda \right)S, \end{array}$$

(3) $$\begin{array}{c} \frac{dE}{dt}=\lambda S-\left(\mu +\rho \right)E, \end{array}$$

(4) $$\begin{array}{c} \frac{dI}{dt}=\rho E-\left(d+\mu +q+\varepsilon \right)I, \end{array}$$

(5) $$\begin{array}{c} \frac{dR}{dt}=qI-\left(\mu +\gamma \right)R, \end{array}$$

(6) $$\begin{array}{c} \frac{dM}{dt}=\varepsilon I-\mu_{M}M, \end{array}$$

with the condition *S* + *E* + *I* + *R* = *N*.

From equation (2), we have

(7) $$\begin{array}{c} \frac{dN}{dt}=\left(b-\mu \right)N-\left(d+\varepsilon \right)I. \end{array}$$

Hence the dynamics of the helminth model can be investigated by normalizing model (2).

Let *s* = *S*/*N*, *e* = *E*/*N*, *i* = *I*/*N*, *r* = *R*/*N*, and *m* = *M*/*K*.

Then, equation (7) can be written in terms of fractional variables as

(8) $$\begin{array}{c} \frac{dN}{dt}=\left(b-\mu -\left(d+\varepsilon \right)i\right)N. \end{array}$$

Using equation (8) and differentiating the fractional variables with respect to *t* leads to the following normalized model:

(9) $$\begin{array}{c} \frac{ds}{dt}=b+\gamma r-\left(b+\frac{\beta m}{1+m}-\left(d+\varepsilon \right)i\right)s, \end{array}$$

(10) $$\begin{array}{c} \frac{de}{dt}=\frac{\beta m}{1+m}s-\left(b+\rho -\left(d+\varepsilon \right)i\right)e, \end{array}$$

(11) $$\begin{array}{c} \frac{di}{dt}=\rho e-\left(b+d+q+\varepsilon -\left(d+\varepsilon \right)i\right)i, \end{array}$$

(12) $$\begin{array}{c} \frac{dr}{dt}=qi-\left(b+\gamma -\left(d+\varepsilon \right)i\right)r, \end{array}$$

(13) $$\begin{array}{c} \frac{dm}{dt}=\varepsilon i-\mu_{M}m. \end{array}$$

The solutions of system (9) enter the region that is positively invariant given by

(14) $$\begin{array}{c} \Omega =\left\{\left(s,e,i,r,m\right)\in {\mathbb{R}}_{+}^{5}\;|\;s+e+i+r\leq 1,0\leq m\leq 1\right\}. \end{array}$$

The following theorem guarantees the well posedness of system (9) such that the solutions with nonnegative initial conditions will remain nonnegative for all future time.

**Theorem 1 .**

The region *Ω* ∈ *ℜ*_+_^5^ is positively invariant with respect to system (9) and nonnegative solutions exist for all future time.

**Proof**

Given the nonnegative initial conditions, it can be shown that the solutions to system (9), *s*(*t*), *e*(*t*), *i*(*t*), *r*(*t*)and*m*(*t*) are non-negative for *t* > 0 otherwise we assume a contradiction that there exists a first time *t*_0_ such that *s*(*t*_0_) = 0, *s*′(*t*_0_) ≤ 0 and *s*(*t*) > 0, *e*(*t*) > 0, *i*(*t*) > 0, *r*(*t*) > 0, *m*(*t*) > 0 for 0 < *t* < *t*_0_; or there exists *t*_1_ such that *e*(*t*_1_) = 0, *e*′(*t*_1_) ≤ 0 and *e*(*t*) > 0, *s*(*t*) > 0, *i*(*t*) > 0, *r*(*t*) > 0, *m*(*t*) > 0 for 0 < *t* < *t*_1_; or there exists *t*_2_ such that *i*(*t*_2_) = 0, *i*′(*t*_2_) ≤ 0 and *i*(*t*) > 0, *s*(*t*) > 0, *e*(*t*) > 0, *r*(*t*) > 0, *m*(*t*) > 0 for 0 < *t* < *t*_2_; or there exists *t*_3_ such that *r*(*t*_3_) = 0, *r*′(*t*_3_) ≤ 0 and *r*(*t*) > 0, *s*(*t*) > 0, *e*(*t*) > 0, *i*(*t*) > 0, *m*(*t*) > 0 for 0 < *t* < *t*_3_; or there exists *t*_4_ such that *m*(*t*_4_) = 0, *m*′(*t*_4_) ≤ 0 and *m*(*t*) > 0, *s*(*t*) > 0, *e*(*t*) > 0, *i*(*t*) > 0, *r*(*t*) > 0 for 0 < *t* < *t*_4_ as in [9].

In the first case, consider the first equation of system (9)

(15) $$\begin{array}{c} \frac{ds\left(t_{0}\right)}{dt}=b+\gamma r\left(t_{0}\right)>0, \end{array}$$

which is a contradiction implying that *s*(*t*) > 0.

In the second case, we have

(16) $$\begin{array}{c} \frac{de\left(t_{1}\right)}{dt}=\frac{\beta m\left(t_{1}\right)s\left(t_{1}\right)}{1+m\left(t_{1}\right)}\geq 0, \end{array}$$

if this is true both *s*(*t*_1_) ≥ 0 and *m*(*t*_1_) ≥ 0. It follows that *e*(*t*_1_) ≥ 0 which is a contradiction implying that *e*(*t*) > 0.

In the third case, we have

(17) $$\begin{array}{c} \frac{di\left(t_{2}\right)}{dt}=\rho e\left(t_{2}\right)>0, \end{array}$$

which is a contradiction implying that *i*(*t*) > 0.

In the fourth case, we have

(18) $$\begin{array}{c} \frac{dr\left(t_{3}\right)}{dt}=\left(q+\tau \right)i\left(t_{3}\right)>0, \end{array}$$

which is a contradiction implying that *r*(*t*) > 0.

In the fifth case, we have

(19) $$\begin{array}{c} \frac{dm\left(t_{4}\right)}{dt}=\varepsilon i\left(t_{4}\right)>0, \end{array}$$

which is a contradiction implying that *m*(*t*) > 0.

Hence, in all cases, *s*(*t*), *e*(*t*), *i*(*t*), *r*(*t*), and *m* are all positive for all *t* > 0. Thus, the components of the solutions to system (9) remain positive for all *t* > 0.

Thus, the helminth model is well posed epidemiologically and mathematically. Thus, it is sufficient to consider the dynamics of (9) in *Ω* [10].

## 3. Model Analysis

The qualitative analysis of the normalized model (9) is considered in order to get an understanding of the dynamics of the helminth infection.

### 3.1. The Disease-Free Equilibrium and Basic Reproduction Number

The disease-free equilibrium of model (9) is obtained by equating all the derivatives to zero and then setting *e* = 0, *i* = 0, *r* = 0, and *m* = 0. Thus, we obtain the DFE as

(20) $$\begin{array}{c} E_{0}=\left(1,0,0,0,0\right). \end{array}$$

The basic reproduction number denoted by *ℛ*_0_ is the quantity that quantifies the ability of the disease to invade the community, and it is the quantity that governs the spread of the disease. Furthermore, it helps in deciding on control strategies to be adopted for disease eradication. Therefore, in our case, *ℛ*_0_ determines the role of parasitic worms/larvae present in the soil to produce secondary infections to the individual hosts.

The next-generation approach is used to obtain *ℛ*_0_ following Diekmann et al. [11]. Using the principles of a next-generation matrix, let

(21) $$\begin{array}{c} F={\left(\frac{\beta m}{1+m}s,0,0,0\right)}^{T},\\ V={\left(\left(b+\rho -\left(d+\varepsilon \right)i\right)e,\left(b+d+q+\tau +\varepsilon -\left(d+\varepsilon \right)i\right)i-\rho e,\left(b+\gamma -\left(d+\varepsilon \right)i\right)r-\left(\tau +q\right)i,\left(\mu_{M}-\theta \right)m-\varepsilon i\right)}^{T}, \end{array}$$

be the appearance of new infections and transfer on individuals between compartments, respectively. Applying the linearization technique, the Jacobian matrices of *ℱ* and *𝒱* at DFE *ℰ*_0_ are

(22) $$\begin{array}{c} F=\left(\begin{array}{cccc} 0 & 0 & 0 & \beta \\ 0 & 0 & 0 & 0\\ 0 & 0 & 0 & 0\\ 0 & 0 & 0 & 0 \end{array}\right),\\ V=\left(\begin{array}{cccc} b+\rho & 0 & 0 & 0\\ -\rho & b+d+q+\varepsilon & 0 & 0\\ 0 & -\left(\tau +q\right) & b+\gamma & 0\\ 0 & -\varepsilon & 0 & \mu_{M} \end{array}\right). \end{array}$$

The basic reproduction number *ℛ*_0_ is the spectral radius of the next-generation matrix *FV*^−1^. Thus,

(23) $$\begin{array}{c} R_{0}=\frac{\rho \varepsilon \beta }{\mu_{M}\left(b+\rho \right)\left(d+b+q+\varepsilon \right)}, \end{array}$$

From (23), the fraction *β*/(*b* + *d* + *q* + *ε*) represents the average number of susceptible individuals being infected during the infectious period, and the fraction *ρ*/(*b* + *ρ*) represents the proportion of individuals that survives the latent period, while *ε*/*μ*_*M*_ represents the fraction of parasites diminished from the environment.

### 3.2. Stability of DFE

We state Theorem 2 for the stability of the DFE.

**Theorem 2 .**

The disease-free equilibrium of model (9) is *LAS ifℛ*_0_ < 1 and unstable if *ℛ*_0_ > 1.

**Proof**

To investigate the local stability of the DFE at *ℰ*_0_, we obtain the Jacobian matrix of equation (9), i.e.,

(24) $$\begin{array}{c} J_{E_{0}}=\left(\begin{array}{ccccc} -b & 0 & -\left(d+\varepsilon \right) & \gamma & -\beta \\ 0 & -\left(b+\rho \right) & 0 & 0 & \beta \\ 0 & \rho & -\left(b+d+q+\varepsilon \right) & 0 & 0\\ 0 & 0 & q & -\left(b+\gamma \right) & 0\\ 0 & 0 & \varepsilon & 0 & -\mu_{M} \end{array}\right). \end{array}$$

Clearly, *λ*_1_ = −*b* and *λ*_2_ = −(*b* + *γ*) are the first and second eigenvalues of the Jacobian matrix *J*_*ℰ*_0__ which are strictly negative. The remaining three can be obtained by considering the submatrix *J*_*ℰ*_0__^1^:

(25) $$\begin{array}{c} J_{E_{0}}^{1}=\left(\begin{array}{ccc} -\left(b+\rho \right) & 0 & \beta \\ \rho & -\left(b+d+q+\varepsilon \right) & 0\\ 0 & \varepsilon & -\mu_{M} \end{array}\right). \end{array}$$

From equation (25), the characteristic equation is given by

(26) $$\begin{array}{c} f\left(\lambda \right)=\lambda^{3}+d_{2}\lambda^{2}+d_{1}\lambda +d_{0}=0, \end{array}$$

where

(27) $$\begin{array}{c} d_{2}=2b+d+q+\rho +\varepsilon +\mu_{M}, \end{array}$$

(28) $$\begin{array}{c} d_{1}=\left(b+\rho \right)\left(b+d+q+\varepsilon \right)+\mu_{M}\left(2b+d+q+\rho +\varepsilon \right), \end{array}$$

(29) $$\begin{array}{c} d_{0}=\mu_{M}\left(b+\rho \right)\left(b+d+q+\varepsilon \right)-\varepsilon \rho \beta . \end{array}$$

Applying the Routh-Hurwitz criteria [12], the conditions in (27) *d*_0_ > 0, *d*_1_ > 0, *d*_2_ > 0, and *d*_2_*d*_1_ > *d*_0_. Here, *d*_1_ > 0, *d*_2_ > 0, and *d*_0_ > 0 when *ℛ*_0_ ≤ 1, and then *d*_2_*d*_1_ > *d*_0_. Hence, according to the Routh-Hurwitz criteria, the submatrix *J*_*ℰ*_0__^1^ has negative real parts whenever *ℛ*_0_ ≤ 1.

Thus, the DFE, *ℰ*_0_ is locally asymptotically stable if *ℛ*_0_ < 1.

### 3.3. Global Stability of the Disease-Free Equilibrium

For the proof of global stability, we consider the theorem developed by Castillo-Chavez et al. [13] and later applied in [14, 15].

To apply the theorem, system (9) is written in the form

(30) $$\begin{array}{c} \frac{dX}{dt}=F\left(X,Z\right), \end{array}$$

(31) $$\begin{array}{c} \frac{dZ}{dt}=G\left(X,Z\right),G\left(X,0\right)=0 \end{array}$$

where *X* = (*s*, *r*) ∈ ℝ_+_^2^ denotes the number of uninfected components (individuals) and *Z* = (*e*, *i*, *m*) ∈ ℝ_+_^3^ denotes infected components (individuals) including the latent and infectious individuals.

The disease-free equilibrium is now denoted by *ℰ*_0_ = (*X*^∗^, 0) = (1, 0).

The conditions (H1) and (H2) in system (32) must be satisfied to guarantee local asymptotic stability:

(32) $$\begin{array}{c} H1:\text{For }\frac{dX}{dt}=F\left(X^{*},0\right),X^{*}\text{ is globally asymptotically stable},\text{ and}\\ H2:G\left(X,Z\right)=AZ-\hat{G}\left(X,Z\right),\hat{G}\left(X,Z\right)\geq 0\text{ for }\left(X,Z\right)\in \Omega , \end{array}$$

where *A* = *D*_*Z*_*G*(*X*^∗^, 0) is the Metzeler-matrix (the off-diagonal elements of *A* are nonnegative) and *Ω* is the region where the model makes biological sense.

If system (30) satisfies the two conditions in (32), then the following theorem holds.

**Theorem 3 .**

The disease fixed point *ℰ*_0_ = (*X*^∗^, 0) is globally asymptotically stable of system (30) provided that *ℛ*_0_ < 1 and that conditions H1 and H2 are satisfied.

**Proof**

From system (9) we have,

(33) $$\begin{array}{c} F\left(X,Z\right)=\left(\begin{array}{c} b+\gamma r-\left(b+\frac{\beta m}{1+m}-\left(d+\varepsilon \right)i\right)s\\ qi-\left(b+\gamma -\left(d+\varepsilon \right)i\right)r \end{array}\right), \end{array}$$

(34) $$\begin{array}{c} G\left(X,Z\right)=\left(\begin{array}{c} \frac{\beta m}{1+m}s-\left(b+\rho -\left(d+\varepsilon \right)i\right)e\\ \rho e-\left(b+d+q+\varepsilon -\left(d+\varepsilon \right)i\right)i\\ \varepsilon i-\mu_{M}m \end{array}\right), \end{array}$$

Then, system (33) can be reduced to

(35) $$\begin{array}{c} {\left.\frac{dX}{dt}\right|}_{Z=0}=\left(\begin{array}{c} b-bs\\ 0 \end{array}\right). \end{array}$$

Now, condition H1 is satisfied as it can be observed from the solution, namely, *s*(*t*) = 1 + (*s*(0) − 1)*e*^−*bt*^. As *t*⟶∞, the solution *s*(*t*)⟶1 implying global convergence of solution of (35) in *Ω*. Thus, condition H1 is satisfied.

Let

(36) $$\begin{array}{c} A=\left(\begin{array}{ccc} -\left(b+\rho \right) & 0 & \beta \\ \rho & -\left(b+d+q+\varepsilon \right) & 0\\ 0 & \varepsilon & -\mu_{M} \end{array}\right), \end{array}$$

Then,

(37) $$\begin{array}{c} \hat{G}\left(X,Z\right)=\left(\begin{array}{c} \hat{G_{1}}\left(X,Z\right)\\ \hat{G_{2}}\left(X,Z\right)\\ \hat{G_{3}}\left(X,Z\right) \end{array}\right)=\left(\begin{array}{c} \left(1-\frac{s}{1+m}\right)\beta m-\left(d+\varepsilon \right)ie\\ -\left(d+\varepsilon \right)i^{2}\\ 0 \end{array}\right). \end{array}$$

From above, $\hat{G}\left(X,Z\right)\geq 0$ if and only if *d* = *ε* = 0. Thus, *ℰ*_0_ may not be globally asymptotically stable for some parameter values. This suggests the possibility of backward bifurcation of Section 3.5.

### 3.4. Existence of Endemic Equilibrium

The endemic equilibrium *ℰ*^∗^ of model (9) is defined as the steady state solution which occurs when disease persists in the community and is obtained by equating the derivatives of system (9) to zero. If we denote the force of infection as *λ*^∗^ = *βm*/1 + *m* at steady states and let *z*^∗^ = *b* − (*d* + *ε*)*i*^∗^ for any choice of *i*^∗^ at the endemic equilibrium; then, the endemic equilibrium can be expressed in terms of the force of infection as

(38) $$\begin{array}{c} s^{*}=\frac{b\left(\gamma +z^{*}\right)\left(\rho +z^{*}\right)\left(d+q+z^{*}+\varepsilon \right)}{\Delta_{3}+{\left(z^{*}\right)}^{3}\left(\lambda^{*}+\Delta_{1}\right)+{\left(z^{*}\right)}^{2}\left(\lambda^{*}\Delta_{1}+\rho \left(\gamma +\Delta_{2}\right)+\gamma \Delta_{2}\right)+z^{*}\Delta_{4}+{\left(z^{*}\right)}^{4}}, \end{array}$$

(39) $$\begin{array}{c} e^{*}=\frac{b\lambda^{*}\left(\gamma +z^{*}\right)\left(\Delta_{2}+z^{*}\right)}{\left(\gamma +z^{*}\right)\left(\lambda^{*}+z^{*}\right)\left(\rho +z^{*}\right)\left(\Delta_{2}+z^{*}\right)-\gamma \lambda^{*}q\rho }, \end{array}$$

(40) $$\begin{array}{c} i^{*}=\frac{b\lambda^{*}\rho \left(\gamma +z^{*}\right)}{\Delta_{3}+{\left(z^{*}\right)}^{3}\left(\lambda^{*}+\Delta_{1}\right)+{\left(z^{*}\right)}^{2}\left(\lambda^{*}\Delta_{1}+\rho \left(\gamma +\Delta_{2}\right)+\gamma \Delta_{2}\right)+z^{*}\Delta_{4}+{\left(z^{*}\right)}^{4}}, \end{array}$$

(41) $$\begin{array}{c} r^{*}=\frac{b\lambda^{*}q\rho }{\Delta_{3}+{\left(z^{*}\right)}^{3}\left(\lambda^{*}+\Delta_{1}\right)+{\left(z^{*}\right)}^{2}\left(\lambda^{*}\Delta_{1}+\rho \left(\gamma +\Delta_{2}\right)+\gamma \Delta_{2}\right)+z^{*}\Delta_{4}+{\left(z^{*}\right)}^{4}}, \end{array}$$

(42) $$\begin{array}{c} m^{*}=\frac{b\lambda^{*}\rho \varepsilon \left(\gamma +z^{*}\right)}{\mu_{M}\left(\Delta_{3}+{\left(z^{*}\right)}^{3}\left(\lambda^{*}+\Delta_{1}\right)+{\left(z^{*}\right)}^{2}\left(\lambda^{*}\Delta_{1}+\rho \left(\gamma +\Delta_{2}\right)+\gamma \Delta_{2}\right)+z^{*}\Delta_{4}+{\left(z^{*}\right)}^{4}\right)}, \end{array}$$

where

(43) $$\begin{array}{c} \Delta_{1}=\gamma +d+q+\rho +\varepsilon ,\\ \Delta_{2}=d+q+\varepsilon ,\\ \Delta_{3}=\gamma \lambda^{*}\rho \left(d+\varepsilon \right),\\ \Delta_{4}=\left(\lambda^{*}\left(\rho \left(\gamma +\Delta_{2}\right)+\gamma \Delta_{2}\right)+\gamma \rho \Delta_{2}\right). \end{array}$$

Using equation five of (38) and the force of infection *λ*^∗^ = *βm*^∗^/(1 + *m*^∗^) with the model parameters, then (38) satisfies the following polynomial:

(44) $$\begin{array}{c} \lambda^{*}P\left(\lambda^{*}\right)=\lambda^{*}\left(A\lambda^{*}+B\right)=0, \end{array}$$

where

(45) $$\begin{array}{c} A=\mu_{M}\left(\gamma \rho \left(d+\varepsilon \right)+z^{*}\left(\rho \left(\gamma +d+q+\varepsilon \right)+\gamma \left(d+q+\varepsilon \right)+z^{*}\left(\gamma +d+q+\rho +z^{*}+\varepsilon \right)\right)\right),\\ B=\mu_{M}z^{*}\left(\gamma +z^{*}\right)\left(\rho +z^{*}\right)\left(d+q+\varepsilon +z^{*}\right)\left(1-R_{0}\right). \end{array}$$

In equation (44), *λ*^∗^ = 0 corresponds to the disease free equilibrium and the polynomial *P*(*λ*^∗^) = 0 corresponds to the existence of endemic equilibrium which co-exist with disease free equilibrium point when *ℛ*_0_ < 1.

### 3.5. Bifurcation Analysis

To investigate the type of bifurcation model (9) exhibits, we use the center manifold theory of Castillo-Chavez and Song [16]. To apply the center manifold theory we make the change of variables for the normalized helminth model (9). Let *x*_1_ = *s*, *x*_2_ = *e*, *x*_3_ = *i*, *x*_4_ = *r*, and *x*_5_ = *m*. Using the vector notation *X* = (*x*_1_, *x*_2_, *x*_3_, *x*_4_, *x*_5_)^*T*^, then (9) can be written in the form *dX*/*dt* = *F*(*x*) with *F* = (*f*_1_, *f*_2_, *f*_3_, *f*_4_, *f*_5_)^*T*^. If we choose *β* = *β*^∗^ as the bifurcation parameter and solving for *ℛ*_0_ = 1 we have:

(46) $$\begin{array}{c} \left(\begin{array}{cc} f_{1} & =b+\gamma x_{4}-\left(b+\frac{\beta^{*}x_{5}}{1+x_{5}}-\left(d+\varepsilon \right)x_{3}\right)x_{1},\\ f_{2} & =\frac{\beta^{*}x_{5}}{1+x_{5}}x_{1}-\left(b+\rho -\left(d+\varepsilon \right)x_{3}\right)x_{2},\\ f_{3} & =\rho x_{2}-\left(b+d+q+\varepsilon -\left(d+\varepsilon \right)x_{3}\right)x_{3},\\ f_{4} & =\left(q+\tau \right)x_{3}-\left(b+\gamma -\left(d+\varepsilon \right)x_{3}\right)x_{4},\\ f_{5} & =\varepsilon x_{3}-\mu_{M}x_{5}, \end{array}\right. \end{array}$$

where

(47) $$\begin{array}{c} \beta^{*}=\frac{\mu_{M}\left(b+\rho \right)\left(d+b+q+\varepsilon +\tau \right)}{\varepsilon \rho }. \end{array}$$

Now, the matrix *W* at DFE *ℰ*_0_ of the equations (46) is

(48) $$\begin{array}{c} W=\left(\begin{array}{ccccc} -b & 0 & -\left(d+\varepsilon \right) & \gamma & -\beta \\ 0 & -\left(b+\rho \right) & 0 & 0 & \beta \\ 0 & \rho & -\left(b+d+q+\varepsilon \right) & 0 & 0\\ 0 & 0 & q & -\left(b+\gamma \right) & 0\\ 0 & 0 & \varepsilon & 0 & -\mu_{M} \end{array}\right), \end{array}$$

At the value *ℛ*_0_ = 1, the Jacobian *W* has a simple zero eigenvalue whose corresponding left and right eigenvectors are expressed as

(49) $$\begin{array}{c} w_{1}=\left(\frac{\mu_{M}\gamma q-\left(b+\gamma \right)\left(\mu_{M}\left(d+\varepsilon \right)+\varepsilon \beta \right)}{b\left(b+\gamma \right)}\right)w_{3},\\ w_{2}=\frac{\beta^{*}\varepsilon w_{3}}{\mu_{M}\left(b+\rho \right)},\\ w_{3}=w_{3}>0\text{ is free},\\ w_{4}=\frac{qw_{3}}{b+\gamma },\\ w_{5}=\frac{\varepsilon w_{3}}{\mu_{M}},\\ \begin{array}{c} v_{1}=v_{4}=0, \end{array}\\ v_{2}=\frac{\rho v_{3}}{b+\rho },\\ v_{3}=v_{3}>0\text{ is free},\\ v_{5}=\frac{\rho \beta^{*}v_{3}}{\mu_{M\left(b+\rho \right)}}. \end{array}$$

Using the result in [16], we need to compute the coefficients *a* and *b* where

(50) $$\begin{array}{c} a=\overset{5}{\underset{k,i,j=1}{\sum }}v_{k}w_{i}w_{j}\frac{\partial^{2}f_{k}}{\partial x_{i}\partial x_{j}}\left(E_{0},\beta^{*}\right),\\ b=\overset{5}{\underset{k,i=1}{\sum }}v_{k}w_{i}\frac{\partial^{2}f_{k}}{\partial x_{i}\partial \beta^{*}}\left(E_{0},\beta^{*}\right) \end{array}$$

Now, evaluating the partial derivatives of system (46) at (*ℰ*_0_, *β*^∗^), we obtain

(51) $$\begin{array}{c} \frac{\partial^{2}f_{1}}{\partial x_{1}\partial x_{5}}=-\beta^{*},\frac{\partial^{2}f_{1}}{\partial x_{3}\partial x_{1}}=d+\varepsilon ,\\ \frac{\partial^{2}f_{2}}{\partial x_{1}\partial x_{5}}=\beta^{*},\\ \frac{\partial^{2}f_{2}}{\partial x_{3}\partial x_{2}}=d+\varepsilon ,\\ \frac{\partial^{2}f_{3}}{\partial x_{3}^{2}}=2\left(d+\varepsilon \right),\\ \frac{\partial^{2}f_{4}}{\partial x_{3}\partial x_{4}}=d+\varepsilon . \end{array}$$

Furthermore,

(52) $$\begin{array}{c} \frac{\partial^{2}f_{1}}{\partial x_{5}\partial \beta^{*}}=-1,\\ \frac{\partial^{2}f_{2}}{\partial x_{5}\partial \beta^{*}}=1. \end{array}$$

Therefore,

(53) $$\begin{array}{c} \begin{array}{c} a=\frac{v_{3}w_{3}^{2}\rho }{b\mu_{M}{\left(b+\rho \right)}^{2}\left(b+\gamma \right)}\varepsilon \beta^{*}a_{0}+2\left(d+\varepsilon \right)v_{3}w_{3}^{2}. \end{array} \end{array}$$

where

(54) $$\begin{array}{c} a_{0}=\left(b+\rho \right)\left[\mu_{M}\gamma q-\left(b+\gamma \right)\left(\mu_{M}\left(d+\varepsilon \right)+\varepsilon \beta^{*}\right)\right]+b\left(b+\gamma \right),\\ b=\frac{\rho \varepsilon \beta^{*}}{\mu_{M}{\left(b+\rho \right)}^{2}}v_{3}w_{3}. \end{array}$$

The coefficient *b* is positive. By Castillo-Chavez and Song [16], coefficient *a* decides the local dynamics of *ℰ*_0_. Therefore, if *a*_0_ < 0, then system (9) will exhibit forward bifurcation, and if *a*_0_ > 0, it undergoes backward bifurcation.

### 3.6. Sensitivity Analysis of the Model Parameters

Numerical sensitivity analysis is done by computing sensitivity indices of basic reproduction number *ℛ*_0_ which measures the model robustness to parameter values [17]. The forward sensitivity index of a variable to a parameter is a ratio of the relative change in the variable to the relative change in the parameter.

**Definition 1 .**

The normalized forward sensitivity index of a variable *u* that depends differentiable on a parameter *ς* is defined as *r*_*ς*_^*u*^ = (*∂u*/*∂ς*).*ς*/*u*.

**Figure 2:**
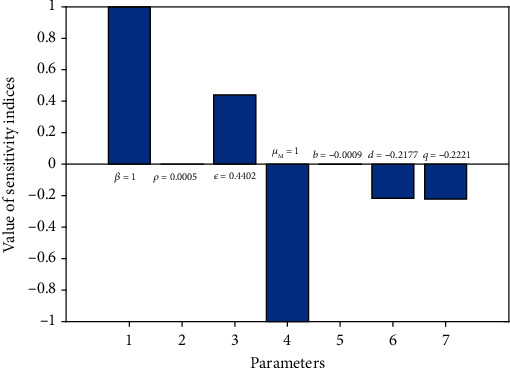
Sensitivity analysis of *ℛ*_0_ with respect to each model parameter.

From equation (23), we compute the sensitivity indices of each parameter involved in *ℛ*_0_ using the equation *r*_*ς*_^*ℛ*_0_^ = (*∂ℛ*_0_/*∂ς*).(*ς*/*ℛ*_0_). For example, the sensitivity index of parameter value with respect to *β* is given by *r*_*β*_^*ℛ*_0_^ = (*∂ℛ*_0_/*∂β*).(*β*/*ℛ*_0_) = 1 and other indices are *r*_*ρ*_^*ℛ*_0_^, *r*_*ε*_^*ℛ*_0_^, *r*_*μ*_*M*__^*ℛ*_0_^, *r*_*b*_^*ℛ*_0_^, *r*_*d*_^*ℛ*_0_^, and *r*_*q*_^*ℛ*_0_^ which were obtained and evaluated using parameter values in Table 1 as illustrated in Figure 2.

To determine the most sensitive parameters for the dynamics of the model, sensitivity analysis was carried out to determine the model robustness to parameter values. Clearly *β*, *ρ*, and *ε* have positive indices with most sensitive parameter be *β* and the least positively sensitive parameter *ρ*. This imply that the endemicity of the disease increases when the parameters *β*, *ρ*, and *ε* are increased while keeping other parameters constant. On the other hand, the parameters *μ*_*M*_, *b*, *d*, and *q* have negative indices and when each of these are decreased, while keeping the other parameters constantly decreases the value of *ℛ*_0_ implying that they decrease the endemicity of the disease. Thus, based on these results, we suggest the following interventions to be made for the eradication of the helminth infection from the endemic communities. The first intervention is the mass cleanliness to reduce the concentration of the parasites from the contaminated environment. The other one is the use of anthelmintic drugs including MDA for both healthy and infected individuals which will in turn reduce the shedding rate of the parasite to the environment.

### 3.7. Extension of the Model to Optimal Control

In this section, we extend the heminth model (2) by incorporating three time-dependent control measures. The best intervention strategies will be identified from combination of these control measures so as to minimize the infection from the community. The optimal control model for helminth infection is formulated by considering the following controls:

1. Health education control *u*_1_(*t*) that is aimed at sensitizing the susceptible population. This is done through provision of education materials (TV messages, radio, posters, and leaflets), educational messages which can be delivered by health practitioners or teachers in schools, education that improve hygiene awareness and behavior of people who defecate outside the latrines, and purchase of shoes to both preschool-aged and school-aged children [18]. Thus. the force of infection is modified as *λ* = ((1 − *κ*_1_*u*_1_)*βM*)/(*K* + *M*) where *κ*_1_ is the effectiveness measure of health education control, where *κ*_1_ ∈ (0, 1) such that if *κ*_1_ = 0, then health education is not effective and *κ*_1_ = 1 corresponds to completely effective health education, 0 < *κ*_1_ < 1 implies that health education is effective to some degree
2. The use of mass drug administration (deworming) *κ*_2_*u*_2_(*t*), which is administered regularly without individual diagnosis is applied to the entire population [19]. Application of this control moves the individuals in exposed class to the susceptible class and those in the infected class to the recovered class
3. Sanitation control (*u*_3_(*t*)) which measures the efforts to prevent susceptible from contracting the disease. This include drinking clean water, personal hygiene, avoiding eating raw vegetables, cleaning of fruits, and intensive cleanliness of the environment which helps to break the helminth transmission cycle (see [19]). Thus, the clearance of the parasite from the environment will be accelerated by *μ*_*M*_ + *κ*_3_*u*_1_, where *κ*_3_ is the effectiveness of the control to protect susceptible population from contracting helminth infection

After incorporating the controls and using the same parameters and variables as in (2), we obtain the modified model with controls for helminth infection given by

(55) $$\begin{array}{c} \frac{dS}{dt}=bN+\gamma R-\mu S-\left(1-\kappa_{1}u_{1}\right)\lambda S+\kappa_{2}u_{2}E, \end{array}$$

(56) $$\begin{array}{c} \frac{dE}{dt}=\left(1-\kappa_{1}u_{1}\right)\lambda S-\left(\mu +\rho \right)E-\kappa_{2}u_{2}E, \end{array}$$

(57) $$\begin{array}{c} \frac{dI}{dt}=\rho E-\left(d+\mu +\varepsilon \right)I-\left(q+\kappa_{2}u_{2}\right)I, \end{array}$$

(58) $$\begin{array}{c} \frac{dR}{dt}=\left(q+\kappa_{2}u_{2}\right)I-\left(\mu +\gamma \right)R, \end{array}$$

(59) $$\begin{array}{c} \frac{dM}{dt}=\varepsilon I-\left(\mu_{M}+\kappa_{3}u_{3}\right)M. \end{array}$$

with *S*(0) = *S*_0_, *E*(0) = *E*_0_, *I*(0) = *I*_0_, *R*(0) = *R*_0_, and *M*(0) = *M*_0_. In this study, we assume that the control functions *u*_1_(*t*), *u*_2_(*t*), and *u*_3_(*t*) are Lebesque integrable functions with 0 ≤ *u*_*i*_ ≤ 1 for *i* = 1, 2, 3 to mean that when the control is zero, there is no any control implemented and when the control is one there is maximum implementation of the control. The objective is to obtain the optimal levels of the control and state variables that optimize the objective functional given by

(60) $$\begin{array}{c} J\left(u_{1},u_{2},u_{3}\right)=\underset{u_{1},u_{2},u_{3}}{\min}\int_{0}^{t_{f}}\left(A_{1}I+A_{2}u_{2}N+\frac{1}{2}\overset{3}{\underset{i=1}{\sum }}\;w_{i}u_{i}^{2}\right)dt. \end{array}$$

Here, we want to minimize the number of infected individual from the population while keeping the cost of controls low. In equation (60), *A*_1_ is the relative weight of the infected individuals that accounts for the social cost while *A*_2_*u*_2_ is the individual cost for MDA of the entire population(preschool- and school-aged children) because it the most vulnerable population for helminth infection. Furthermore, *w*_*i*_ is the relative costs weight associated with background costs for each control measure *u*_*i*_ such as advisement costs, production of posters, ordering and transportation of drugs and water treatment and *t*_*f*_ is the final fixed time. In this work, the cost is not linear as may result to bang bang which means that the optimal control take values at only upper and lower control set; thus, we choose to have quadratic cost on the control of the form (1/2)*w*_*i*_*u*_*i*_^2^ (see [20]). The problem becomes of determining the optimal triplet (*u*_1_^∗^, *u*_2_^∗^, *u*_3_^∗^) such that

(61) $$\begin{array}{c} J\left(u_{1}^{*},u_{2}^{*},u_{3}^{*}\right)=\underset{u_{1},u_{2},u_{3}\in U}{\min}J\left(u_{1},u_{2},u_{3}\right), \end{array}$$

where *U* = {(*u*_1_, *u*_2_, *u*_3_) | each *u*_*i*_ is measurable with 0 ≤ *u*_*i*_ ≤ 1 for 0 ≤ *t* ≤ *t*_*f*_} is the admissible set.

#### 3.7.1. Existence of an Optimal Control

The solution of the helminth model (9) is proved to be bounded so we use this result to prove the existence of the optimal control. However, existence of optimal control is shown using the approach of Fleming and Richel [14] and later applied in [15, 21–23]. For detailed proof, (see [14], Theorem 4.1, p 68-69).

#### 3.7.2. The Hamiltonian and Optimality Systems

To obtain the Hamiltonian *H*, we apply “Pontryagin's maximum principle” [24] to derive the necessary conditions for the optimal pair. Therefore, the Hamiltonian is expressed as

(62) $$\begin{array}{c} H\left(S,E,I,R,M,t\right)=A_{1}I+A_{2}u_{2}N+\frac{1}{2}\overset{3}{\underset{i=1}{\sum }}w_{i}u_{i}^{2}+\lambda_{1}\frac{dS}{dt}+\lambda_{2}\frac{dE}{dt}+\lambda_{3}\frac{dI}{dt}+\lambda_{4}\frac{dR}{dt}+\lambda_{5}\frac{dM}{dt}, \end{array}$$

with *λ*_*i*_, *i* = 1, 2, 3, 4, 5 denotes the adjoint variables to be determined by taking the negative derivative of the Hamiltnian function.

**Theorem 4 .**

Given the optimal set u_1_, u_2_, and u_3_ that minimizes J over U, there exist an adjoint variables *λ*_1_, *λ*_2_, *λ*_3_, *λ*_4_, and *λ*_5_ such that

(63) $$\begin{array}{c} \frac{d\lambda_{1}}{dt}=-A_{2}u_{2}+\frac{\left(1-\kappa_{1}u_{1}\right)\beta M}{K+M}\left(\lambda_{1}-\lambda_{2}\right)+\left(\mu -b\right)\lambda_{1}, \end{array}$$

(64) $$\begin{array}{c} \frac{d\lambda_{2}}{dt}=-A_{2}u_{2}+\left(\mu +\rho \right)\lambda_{2}+\kappa_{2}u_{2}\left(\lambda_{2}-\lambda_{1}\right)-b\lambda_{1}-\rho \lambda_{3}, \end{array}$$

(65) $$\begin{array}{c} \frac{d\lambda_{3}}{dt}=-A_{1}-A_{2}u_{2}-b\lambda_{1}+\left(d+\mu +\varepsilon \right)\lambda_{3}+\left(q+\kappa_{2}u_{2}\right)\left(\lambda_{3}-\lambda_{4}\right)-\varepsilon \lambda_{5}, \end{array}$$

(66) $$\begin{array}{c} \frac{d\lambda_{4}}{dt}=-A_{2}u_{2}-\left(b+\gamma \right)\lambda_{1}+\left(\mu +\gamma \right)\lambda_{4}, \end{array}$$

(67) $$\begin{array}{c} \frac{d\lambda_{5}}{dt}=\left(\frac{\left(1-\kappa_{1}u_{1}\right)S\beta }{K+M}-\frac{\left(1-\kappa_{1}u_{1}\right)SM\beta }{{\left(K+M\right)}^{2}}\right)\left(\lambda_{1}-\lambda_{2}\right)+\left(\mu_{M}+\kappa_{3}u_{3}\right)\lambda_{5}, \end{array}$$

with transversality conditions, *λ*_*i*_(*t*_*f*_) = 0, *i* = 1, 2, 3, 4, 5. The characterization of the control set (*u*_1_^∗^, *u*_2_^∗^, and *u*_3_^∗^) using the technique of control bounds gives

(68) $$\begin{array}{c} u_{1}^{*}\left(t\right)=\max\left\{0,\min\left(1,\Phi_{1}\right)\right\},\\ u_{2}^{*}\left(t\right)=\max\left\{0,\min\left(1,\Phi_{2}\right)\right\},\\ u_{3}^{*}\left(t\right)=\max\left\{0,\min\left(1,\Phi_{3}\right)\right\}, \end{array}$$

where

(69) $$\begin{array}{c} \Phi_{1}=\frac{\kappa_{1}\beta MS}{w_{1}\left(K+M\right)}\left(\lambda_{2}-\lambda_{1}\right),\\ \Phi_{2}=\frac{\kappa_{2}E\left(\lambda_{2}-\lambda_{1}\right)+\kappa_{2}I\left(\lambda_{3}-\lambda_{4}\right)}{w_{2}}-\frac{A_{2}\left(S+E+I+R\right)}{w_{2}},\\ \Phi_{3}=\frac{\kappa_{3}M\lambda_{5}}{w_{3}}. \end{array}$$

**Proof**

By using ([24]), the adjoint system is the Euler-Lagrange equations obtained taking the negative of the partial derivative of the Hamiltonian in (62) with respect to the state variables. Thus, the adjoint system can be written as

(70) $$\begin{array}{c} \frac{d\lambda_{1}}{dt}=-\frac{\partial H}{dS}=-A_{2}u_{2}+\frac{\left(1-\kappa_{1}u_{1}\right)\beta M}{K+M}\left(\lambda_{1}-\lambda_{2}\right)+\left(\mu -b\right)\lambda_{1},\\ \frac{d\lambda_{2}}{dt}=-\frac{\partial H}{dE}=-A_{2}u_{2}+\left(\mu +\rho \right)\lambda_{2}+\kappa_{2}u_{2}\left(\lambda_{2}-\lambda_{1}\right)-b\lambda_{1}-\rho \lambda_{3},\\ \frac{d\lambda_{3}}{dt}=-\frac{\partial H}{dI}=-A_{1}-A_{2}u_{2}-b\lambda_{1}+\left(d+\mu +\varepsilon \right)\lambda_{3}+\left(q+\kappa_{2}u_{2}\right)\left(\lambda_{3}-\lambda_{4}\right)-\varepsilon \lambda_{5},\\ \frac{d\lambda_{4}}{dt}=-\frac{\partial H}{dR}=-A_{2}u_{2}-\left(b+\gamma \right)\lambda_{1}+\left(\mu +\gamma \right)\lambda_{4},\\ \frac{d\lambda_{5}}{dt}=-\frac{\partial H}{dM}=\left(\frac{\left(1-\kappa_{1}u_{1}\right)S\beta }{K+M}-\frac{\left(1-\kappa_{1}u_{1}\right)SM\beta }{K+M^{2}}\right)\left(\lambda_{1}-\lambda_{2}\right)+\left(\mu_{M}+\kappa_{3}u_{3}\right)\lambda_{5}. \end{array}$$

Using the same principle, we get the controls by solving the equation obtained by taking the partial derivative of the Hamiltonian with respect to each control. For example,

(71) $$\begin{array}{c} \frac{\partial H}{\partial u_{1}}=w_{1}u_{1}+\frac{\kappa_{1}\beta MS}{K+M}\lambda_{1}-\frac{\kappa_{1}\beta MS}{K+M}\lambda_{2},\\ \frac{\partial H}{\partial u_{2}}=w_{2}u_{2}+\kappa_{2}E\left(\lambda_{1}-\lambda_{2}\right)+\kappa_{2}I\left(\lambda_{3}-\lambda_{4}\right),\\ \frac{\partial \text{H}}{\partial u_{3}}=w_{3}u_{3}-\kappa_{3}M\lambda_{5}. \end{array}$$

Now, setting *∂H*/*∂u*_*i*_ = 0 at *u*_1_^∗^ for *i* = 1, 2, 3, we obtain the controls set:

(72) $$\begin{array}{c} u_{1}^{*}\left(t\right)=\frac{\kappa_{1}\beta MS}{w_{1}\left(K+M\right)}\left(\lambda_{2}-\lambda_{1}\right),\\ u_{2}^{*}\left(t\right)=\frac{\kappa_{2}E\left(\lambda_{2}-\lambda_{1}\right)+\kappa_{2}I\left(\lambda_{3}-\lambda_{4}\right)}{w_{2}}-\frac{A_{2}\left(S+E+I+R\right)}{w_{2}},\\ u_{3}^{*}\left(t\right)=\frac{\kappa_{3}M\lambda_{5}}{w_{3}}. \end{array}$$

The controls can then be written using standard control arguments with bounds on the controls as

(73) $$\begin{array}{c} \begin{array}{c} u_{1}^{*}=\left(\begin{array}{c} \Phi_{1}\text{ if }0<\Phi_{1}<1,\\ 0\text{ if }\Phi_{1}\leq 0,\\ 1\text{ if }\Phi_{1}\geq 1. \end{array}\right. \end{array}\\ \begin{array}{c} u_{2}^{*}=\left(\begin{array}{c} \Phi_{2}\text{ if }0<\Phi_{2}<1,\\ 0\text{ if }\Phi_{2}\leq 0,\\ 1\text{ if }\Phi_{2}\geq 1. \end{array}\right. \end{array}\\ \begin{array}{c} u_{3}^{*}=\left(\begin{array}{c} \Phi_{3}\text{if }0<\Phi_{3}<1,\\ 0\text{ if }\Phi_{3}\leq 0,\\ 1\text{ if }\Phi_{3}\geq 1. \end{array}\right. \end{array} \end{array}$$

In compact notation, we writ,

(74) $$\begin{array}{c} u_{1}^{*}\left(t\right)=\max\left\{0,\min\left(1,\Phi_{1}\right)\right\},\\ u_{2}^{*}\left(t\right)=\max\left\{0,\min\left(1,\Phi_{2}\right)\right\},\\ u_{3}^{*}\left(t\right)=\max\left\{0,\min\left(1,\Phi_{3}\right)\right\}. \end{array}$$

where

(75) $$\begin{array}{c} \Phi_{1}=\frac{\kappa_{1}\beta MS}{w_{1}\left(K+M\right)}\left(\lambda_{2}-\lambda_{1}\right),\\ \Phi_{2}=\frac{\kappa_{2}E\left(\lambda_{2}-\lambda_{1}\right)+\kappa_{2}I\left(\lambda_{3}-\lambda_{4}\right)}{w_{2}}-\frac{A_{2}\left(S+E+I+R\right)}{w_{2}},\\ \Phi_{3}=\frac{\kappa_{3}M\lambda_{5}}{w_{3}}. \end{array}$$

The optimality system consists of the state system (55) coupled with adjoint system (63) together with the characterization of the optimal control with the initial and transversality conditions.

The state equations in (55) and the costate (adjoint) equation in system (63) are bounded and satisfies the Lipchitz condition, thus uniqueness of the optimality systems follows using the approaches in [25, 26].

### 3.8. Numerical Simulation

We perform numerical simulation of the optimal control model using the parameter values given in Table 1. Various optimal control strategies are applied to control the burden of helminth infection in the community. Starting with an initial guess for the optimal controls *u*_1_(*t*), *u*_2_(*t*), and *u*_3_(*t*), state system (55) is solved numerically forward in time using the fourth-order Runge-Kutta method with initial conditions *S*(0) = 1000, *E*(0) = 150, *I*(0) = 50, *R*(0) = 5, and *M*(0) = 200. Then, co state (adjoint) system (63) is solved numerically backward in time using the fourth-order Runge-Kutta method with the state variables and initial guess of the controls obtained earlier. All controls are updated using convex combination of the previous controls and its characterization then the process is repeated until convergence is achieved. The following cost weights were used in the simulation: *A*_1_ = 800, *A*_2_ = 0.15, *w*_1_ = 15, *w*_2_ = 30, and *w*_3_ = 10 with the efficacy rates *κ*_1_ = 0.7, *κ*_2_ = 0.8, and *κ*_3_ = 0.6.

#### 3.8.1. Control with Health Education and MDA

**Figure 3:**
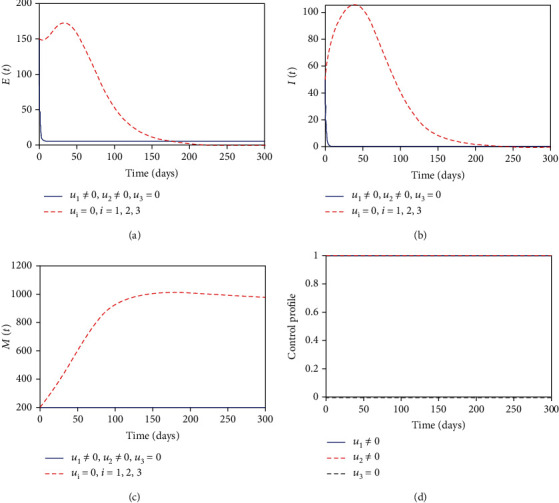
Simulations of the optimal control model with health education and MDA interventions.

In this strategy, we used the combination of health education and MDA interventions to optimize the objective functional *J* while sanitation control is set to zero.  Figure 3(a) shows that with this strategy there is a significant effect in reducing the number of exposed individuals compared with the case when there is no control but does not bring the exposed individuals to zero at the final control period. The helminth-infected individuals in Figure 3(b) decreases to almost zero when the optimal combination of health education and MDA are in place compared to when there is no control. In Figure 3(c), we observed that the parasite population in the environment is reduced but does not reach zero at the final control period. This suggests that using combination of control measures, the infection can be lowered in the community but not completely removed in the specified control period. The control profile in Figure 3(d) suggests that control combination with health education and MDA should be maximized and applied throughout the intervention period.

#### 3.8.2. Control with MDA and Sanitation

**Figure 4:**
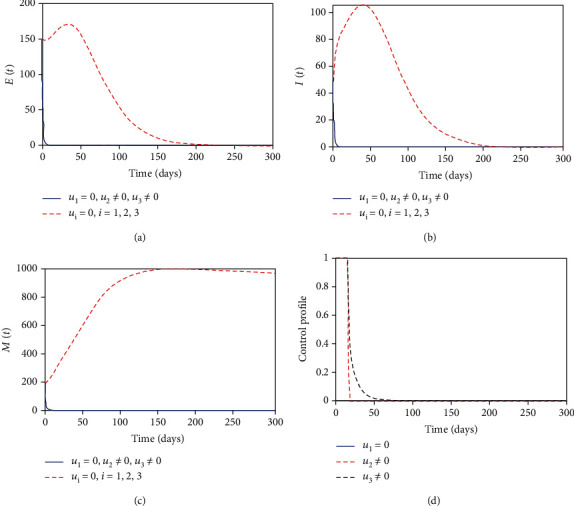
Simulations of the optimal control model with MDA and sanitation interventions.

Next, we consider the combination of MDA and sanitation interventions while the health education is set to zero. Figures 4(a) and 4(b) show a similar trend as in Figures 3(a) and 3(b), respectively, but with this strategy more effective than the combination of health education and MDA. This is due to the fact that with MDA, anthelminthic drugs are regularly administered to the entire population (preschool- and school-aged children) while using sanitation clears the density of parasites from the environment. The control profile in Figure 4 suggests the control with MDA should be seized after 20 days while sanitation control should be kept at a maximum for approximately 20 days and then decreased to zero. Therefore, these results show that combination of these two controls measures can as well manage to control helminth infection in the community.

#### 3.8.3. Control with Health Education and Sanitation

**Figure 5:**
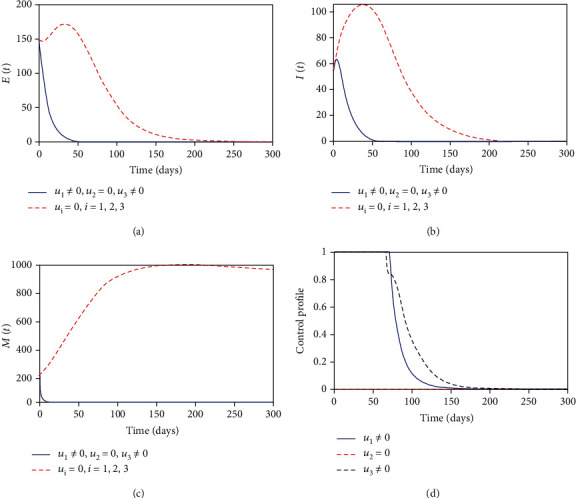
Simulations of the optimal control model with health education and sanitation interventions.

We next consider the control with combination of health education and sanitation in the absence of MDA. The health education and sanitation were used to optimize the objective functional *J* while MDA control was set to zero.  Figure 5(a) shows that the number of exposed individuals were reduced and were totally controlled after 50 days.  Figure 5(b) shows that the number of infected individuals were reduced and totally controlled at the final period.  Figure 5(c) shows that the parasite population is reduced to the minimum level. The control profile in Figure 5(d) suggests that the control with health education should be maximized for 70 days while sanitation should be maximized for 65 days.

#### 3.8.4. Control with Health Education, MDA, and Sanitation

**Figure 6:**
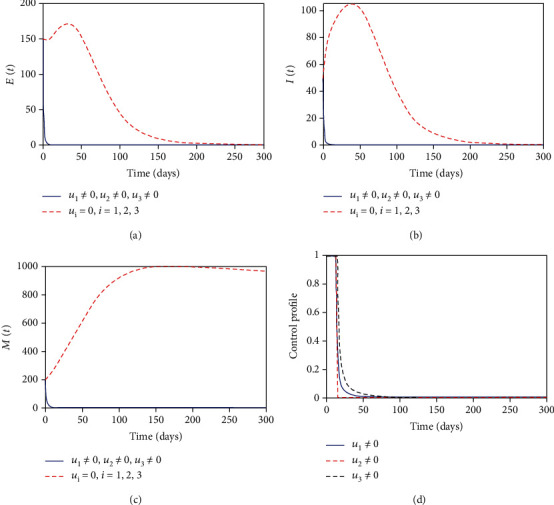
Simulations with health education, MDA, and sanitation interventions.

Finally, we consider the case where all controls were in place. In this strategy the controls' health education, MDA, and sanitation were used to optimize the objective functional *J*.  Figure 6(a) shows that the number of exposed individuals is totally controlled at the final control period.  Figure 6(b) shows that the number of helminth-infected individuals is also totally controlled at the final control period.  Figure 6(c) shows that the parasite population is more reduced to zero at the final control time.  Figure 6(d) suggests that the health education control should be maximized for 15 days, the MDA control be seized after 15 days, while the sanitation should be maximized for 18 days and reduced to zero due to the fact that the infection has been cleared in the community.

### 3.9. Cost-Effectiveness Analysis

Cost effectiveness analysis is performed to quantify the cost effectiveness of different combinations of control strategies using Incremental Cost Effective Ratio (ICER). In this approach, when comparing two competing intervention strategies, one intervention is compared with the next less effective alternative [28, 30, 31]. The ICER formula is given by

(76) $$\begin{array}{c} \text{ICER}=\frac{\text{Difference in costs between strategies}}{\text{Difference in the totalnumber of infection averted}}. \end{array}$$

To obtain the total number of infections averted, we take the sum of the difference between the total number of exposed and infected individuals without and with control. Also to obtain the total cost for each strategy, we used the cost function as shown in Table 2.

Comparing strategy 3.8.1 and 3.8.4, strategy 3.8.1 is more costly and less effective than strategy 3.8.4. Hence, we omit strategy 3.8.1 and recompute ICER values as indicated in Table 3.

From Table 3, we observe that strategy 3.8.2 has higher cost compared to strategy 3.8.4, Hence, we omit 3.8.2 and recompute ICER as indicated in Table 4.

From Table 4, we conclude that strategy 3.8.3 (control with health education and sanitation) is more cost effective than strategy 3.8.4 (control with health education, MDA and sanitation), implying that it is the cheapest of all the control strategies.

## 4. Discussions and Conclusions

In this paper, we formulated and analysed deterministic model for the helminth infection (soil-transmitted helminth).

In Section 3, we analysed the model by obtaining the feasible region and disease-free and endemic equilibrium points with their stability. Moreover, bifurcation analysis were carried out and the model exhibited backward bifurcation for some parameter values, and sensitivity analysis of *ℛ*_0_ with respect to parameters *β*, *ρ*, *ε*, *μ*_*M*_, *b*, *d*, *q* was done and results revealed that *β* was the most positive sensitive parameter while *μ*_*M*_ was the most negative sensitivity parameter. Furthermore, the helminth model developed in Section 2 was extended to include health education, treatment by mass drug administration and sanitation as control measures. We solved the optimal control problem using Pontryagin's maximum principle by first obtaining the Hamiltonian, the adjoint variables, and the characterization of the controls. Applying single-control measure (health education or MDA or sanitation) alone is not sufficient to control the infection; thus, combination of control measures were used to optimize the objective functional *J* by considering combination of health education with mass drug administration, combination of mass drug administration with sanitation, combination of health education with sanitation, and all the three controls. Finally, we investigated the cost effectiveness of the control strategies to ascertain the least and most expensive strategies. Based on our results, we conclude that the combination of health education and sanitation is the best strategy.

## Figures and Tables

**Table 1:** Parameter values for soil-transmitted helminth infection.

Parameter	Symbol	Value	Source
Per capita birth rate	*b*	$\frac{1}{18250}\text{da}{\text{y}}^{-1}$	[27]
Per capita mortality rate	*μ*	$\frac{1}{\left(60\times 365\right)}\text{da}{\text{y}}^{-1}$	[28]
Disease induced death rate	*d*	$\frac{35}{1000}\text{da}{\text{y}}^{-1}$	[27]
Ingestion rate of parasites	*β*	7 parasites day^−1^	Assumed
Carrying capacity of parasite in the environment	*K*	10^5^ parasites	Assumed
Natural recovery rate	*q*	$\frac{1}{28}{\text{day}}^{-1}$	Assumed
Efficacy for MDA drug	*κ*_2_,	0.8	[29]
Immunity waning rate for recovered individuals	*γ*	$\frac{1}{7}\text{da}{\text{y}}^{-1}$	[27]
Clearance rate for parasitic worms	*μ*_*M*_	$\frac{13}{37500}\text{da}{\text{y}}^{-1}$	[27]
Shading rate of parasites to the environment	*ε*	0.09 day^−1^	Assumed
Progression rate from exposed to infected class	*ρ*	$\frac{1}{10}\text{da}{\text{y}}^{-1}$	[27]

**Table 2:** Ranking of control strategies in ascending order of the number of infections averted.

Strategy	Total infection averted	Total costs ($)
No strategy	0	0
Strategy 3.8.1	8.0089 × 10^4^	7.8901 × 10^5^
Strategy 3.8.4	8.0092 × 10^4^	9.9591 × 10^3^
Strategy 3.8.2	8.0098 × 10^4^	1.1149 × 10^4^
Strategy 3.8.3	8.0176 × 10^4^	3.3270 × 10^3^

ICER(3.8.1) = 7.8901 × 10^5^/8.0089 × 10^4^ = 9.85, ICER(3.8.4) = 9.9591 × 10^3^ − 7.8901 × 10^5^/8.0092 × 10^4^ − 8.0089 × 10^4^ = −259683.6, ICER(3.8.2) = 1.1149 × 10^4^ − 9.9591 × 10^3^/8.0098 × 10^4^ − 8.0092 × 10^4^ = 225.15, ICER(3.8.3) = 3.3270 × 10^3^ − 1.1149 × 10^4^/8.0176 × 10^4^ − 8.0098 × 10^4^ = −100.28.

**Table 3:** Computation of ICER after omitting strategy 3.8.1.

Strategy	Total infection averted	Total costs $	ICER
Strategy 3.8.4	8.0092 × 10^4^	9.9591 × 10^3^	-259,683.6
Strategy 3.8.2	8.0098 × 10^4^	1.1149 × 10^4^	225.15
Strategy 3.8.3	8.0176 × 10^4^	3.3270 × 10^3^	-100.28

**Table 4:** Computation of ICER after omitting strategy 3.8.2.

Strategy	Total infection averted	Total costs ($)	ICER
Strategy 3.8.4	8.0092 × 10^4^	9.9591 × 10^3^	0.12
Strategy 3.8.3	8.0176 × 10^4^	3.3270 × 10^3^	-100.28

## Data Availability

The data are available inside the manuscript and were obtained from related published articles. There is no restriction on the availability of the data.

## Supplementary Materials

Supplementary Materials The supplementary material contains the MatLab codes used for numerical simulations for sensitivity analysis and optimal control problem.
